# Clergy-Perpetrated Sexual Abuse in Ghana: A Media Content Analysis of Survivors, Offenders, and Offence Characteristics

**DOI:** 10.1007/s10943-021-01430-3

**Published:** 2021-09-20

**Authors:** Emmanuel Nii-Boye Quarshie, Priscilla Ayebea Davies, Jeremiah Wezenamo Acharibasam, Christiana Owiredua, Prince Atorkey, Daniel Annang Quarshie, Sandra Naa-Shasha Quarshie

**Affiliations:** 1 Faculty of Medicine and Health, School of Psychology, University of Leeds, Leeds, LS2 9JT West Yorkshire UK; 2 Department of Psychology, University of Ghana, Accra, Ghana; 3 Centre for Suicide and Violence Research, Accra, Ghana; 4 Pentecost University College, Accra, Ghana; 5 Department of Community Health and Epidemiology, University of Saskatchewan, Saskatoon, Canada; 6 School of Law, Psychology and Social Work, Örebro University, Örebro, Sweden; 7 School of Medicine and Public Health, University of Newcastle, Callaghan, Australia; 8 The Methodist Church Ghana, Lawra, Upper West Region, Ghana; 9 Ledzokuku-Krowor Municipal Assembly Polyclinic, Accra, Ghana

**Keywords:** Clergy, Clergy-perpetrated sexual abuse, Ghana, Neo-prophetic ministry, Sexual abuse

## Abstract

While there are no official data and published studies on clergy-perpetrated sexual abuse (CPSA) from Ghana, local media reports continue to show worrying trends of the phenomenon. We drew on 73 media reports from January 2000 to March 2019, to describe the offence characteristics and profiles of the perpetrators and survivors of CPSA in Ghana. The findings showed females aged 10–19 as predominant survivors. The perpetrators were all males found guilty of lone rape, incest, defilement, indecent assault, sodomy, attempted rape, or gang rape. A preventive measure could involve streamlining the recruitment, training, and leadership structures of the church.

## Introduction

Sexual abuse and misconduct by the clergy is a global problem and not regarded as a recent phenomenon (Garland & Argueta, 2010; Swain, 2018). Generally, religion provides directives for human welfare and positive action (Bottoms et al., 2004) and religious leaders are usually the first point of contact for their followers experiencing any form of personal crisis or social discomfort (Cameron, 2000; Taylor et al., 2000). Churches have a longstanding foundation of care and providing a safe and supportive environment that enhances and promotes healthful behavioural changes among congregants, while congregational personal relationships with the clergy are founded on principles of truth, trust, respect, and support that provide a promising opportunity for growth and improved physical, emotional, and spiritual health (Cameron, 2000; Peterson et al., 2002; Taylor et al., 2000). However, there is considerable public concern about the violations of religious, professional, and ethical boundaries by some religious leaders who sexually abuse congregants and supplicants, including children (Firestone et al., 2009; UNICEF, 2014). Currently, there is increasing political and media attention on sexual offences in—Christian—religious settings (Death, 2016, 2019; Denney et al., 2018; Lonne & Parton, 2014). Available studies have reported various characteristics and profiles of clergy-perpetrated sexual abuse (CPSA).

### Survivors of CPSA

The survivors/victims of CPSA vary, ranging from children to adults. Girls are three times more likely to be abused than boys in the general population of young people (Sedlak & Broadhurst, 1996; UNICEF, 2014). However, the gender differences in the survivors of CPSA vary, depending on the category of offending clergies. Where the clergies have no stringent rules on celibacy, females are generally found to be the victims/survivors of CPSA (Fogler, et al., 2008a, 2008b; Francis & Baldo, 1998). However, males are the most frequently sexually abused where church policies on celibacy are strictly enforced (Firestone et al., 2009; Fogler, et al., 2008a, 2008b). The evidence suggests further that male survivors of CPSA are generally young boys under age 17 (Firestone et al., 2009; Parkinson et al., 2012; Terry et al., 2011). The John Jay College of Criminal Justice reports that about 51% of the survivors of sexual abuse perpetrated by catholic priests are between the ages of 11 and 14 years (The John Jay College of Criminal Justice, 2004). However, in studies where the survivors are usually females, the age distribution cuts across childhood through adulthood; in other words, whereas perpetrators of CPSA target young males, females are abused regardless of their age (Fogler, et al., 2008a, 2008b; Francis & Baldo, 1998; Friberg & Laaser, 1998). Thus, the survivors of CPSA are more likely to be females, in congregations where leaders of Christian denominations are not bound by any policy of celibacy (Chaves & Garland, 2009; Frame, 1996). For example, in the Church of England, 24% of the priests have reported that since entering ministry have engaged in sexually inappropriate acts with adults who were not their spouses (Birchard, 2000).

On how the perpetrators choose their victims, there is evidence to suggest that the selection is based mainly on availability and access (Holt & Massey, 2013). This could be the reason Catholic priests who serve in dioceses have a higher number of youthful victims compared to priests who serve in orders. This is because diocesan priests are in frequent contact with the (potential) victims, while order priests have limited contact with the outside context (The John Jay College of Criminal Justice, 2004).

### Perpetrators of CPSA

Studies have consistently shown clergymen or male leaders who serve in various capacities in the Christian church as the usual offenders in CPSA (Francis & Baldo, 1998; Friberg & Laaser, 1998; Garland & Argueta, 2010). This is to be expected as the leadership positions/roles in Christian churches and other religious groups are predominantly occupied/played by males. Most Christian religious groups and belief systems in sub-Saharan Africa, for example, “favour” male leadership, while some prohibit females from holding top leadership positions (Agadjanian, 2015; Mhando et al., 2018). Thus, it is not entirely surprising that perpetrators of CPSA are typically males. Globally, (young) males have been found to be the predominant perpetrators of sexual offences against, mostly, girls and women (UNICEF, 2014, 2020; WHO, 2002).

Additionally, offenders in CPSA have commonly been found to be unmarried, never married, and in their 30 s and 40 s (Firestone et al., 2009; Plante & Aldridge, 2005; Terry, 2008; Terry et al., 2011). Although some earlier studies indicate that offenders in CPSA are mostly married (Francis & Baldo, 1998; Friberg & Laaser, 1998), the evidence also suggests that clergymen who are themselves survivors of sexual abuse as children are more likely to be perpetrators of sexual abuse later in life as adults or priests (Terry, 2008; Terry et al., 2011). Moreover, many offenders in CPSA have been found to abuse more than one victim (Friberg & Laaser, 1998; Terry, 2008) and are often in full time priestly or pastoral service (Francis & Baldo, 1998).

### Other Characteristics of CPSA

The commonly reported location where CPSA occurs is the offender’s residence or a private location chosen by the offender, and the offence typically involves fondling, fellatio, kissing, touching, and penetrating the victim (Denney et al., 2018; Firestone et al., 2009; Isely et al., 2008; Terry et al., 2011). The offence also occurs often at a religious facility (in the church building), but perpetrators with multiple victims are more likely to carry out the offence in the perpetrators’ own residence. Sometimes, CPSA are committed because of the lack of supervision of junior clerics by those higher up, for example, bishops (Terry, 2008; Terry et al., 2011; The John Jay College of Criminal Justice, 2004).

Like most sexual offences, the medico-legal effects of CPSA are varied and telling on both offenders and survivors. Whereas offenders face legal prosecution and punishment (including prison terms), the effects of CPSA on the survivors could be long-term, involving both psychological (e.g. post-traumatic stress disorders, depression) and physical/medical health problems—for example, genital and gynaecological injuries and sexually transmitted infections (Carr et al., 2020; UNICEF, 2014; WHO, 2003). Male survivors of sexual abuse also experience similar physical and psychological impacts—fear, depression, suicidal ideations, and anger (WHO, 2003).

### CPSA in Ghana

Evidence from primary studies and systematic reviews have shown that most of what we know about the phenomenon of CPSA is based on published evidence of studies conducted in high-income countries (Astbury, 2013; Blakemore et al., 2017; Denney et al., 2018; Fogler, et al., 2008a, 2008b; Fogler, et al., 2008a, 2008b; Herbert et al., 2020; Lusky-Weisrose et al., 2020; The John Jay College of Criminal Justice, 2004). Little is still known about the phenomenon in low- and middle-income contexts, such as sub-Saharan Africa, where most countries—including Ghana—are considered highly religious (Agazue, 2016; Ayodele, 2019; Chivasa, 2017).

Ghana is a religious Western sub-Saharan African country: 71.2% of the population is Christian, 17.6% Muslim, and about 5.2% have indigenous religious affiliations (Ghana Statistical Service, 2013). Christian ministries and churches are widespread across the various regions of the country. Besides the 1992 Constitution of Ghana providing the basic regulatory framework for the formation and operations of religious groups and expression of religious beliefs, the Ghana Catholic Bishops’ Conference (GCBC), the Christian Council of Ghana (CCG), and the Ghana Pentecostal and Charismatic Council (GPCC) are the three main bodies that seek to provide leadership and good governance of Christian religious groups and churches in the country. Churches affiliated with the GCBC and the CCG are the historical mainline mission churches—for example, Roman Catholic, Methodist, and the Presbyterian Church. Currently, there are 29 local churches affiliated with the CCG, while 230-member churches are affiliated with the GPCC (Christian Council of Ghana, 2019; Ghana Pentecostal & Charismatic Council, 2019; Ghana Statistical Service, 2013).

Recently, a new strand of churches or Christian Ministries known as Neo-prophetic churches has also emerged—detailed discussion of neo-prophetic churches in Ghana has been provided elsewhere (Omenyo, 2011; Omenyo & Arthur, 2013).

To date, we are not aware of any published study on CPSA in Ghana—there are no official data or police recorded statistics specifically on the phenomenon in the country. Recent frequent local media reports and anecdotal evidence on CPSA have generated considerable public concern; thus, the present study is born out of the need for systematic evidence on the phenomenon in Ghana. The aim of this study is to explore, through content analysis of local Ghanaian media reports, some of the key socio-demographic profiles and commonly reported characteristics of the survivors/victims, offenders, and the offence of CPSA in Ghana. We hope that the evidence presented by this study would represent a useful point of departure for further primary research on the phenomenon in Ghana to inform intervention and prevention efforts in the country.

## Methods

### Research Approach and Data Source

We used the media content analysis approach for this study (Macnamara, 2005). Media content analysis is a variant of content analysis that is used to study a wide range of textual data drawn from various media contents: news, films, editorials, television programmes, and advertisements contained in magazines, online portals, and newspapers (Macnamara, 2005; Neuendorf, 2002; Riffe et al., 2014). Specifically, we modelled this study after recent studies using media content analysis approach to systematically explore issues and topics that had not been previously researched in Ghana, for example, multiple perpetrator rape (Quarshie et al., 2018) and incest (Quarshie et al., 2017). More recently, media content analysis has been found useful in the study of child sexual abuse in Christian congregations in Nigeria (Agazue, 2016) and the USA (Denney et al., 2018). Thus, the data for the present study comprised a collection of online media reports on CPSA drawn from the online portals of four categories of media outlets in Ghana: television (*TV3* and *GTV*), radio (*Joy FM, Kasapa FM, Citi FM, STARR FM, Peace FM,* and *3News*), newspaper (*Daily Guide, Daily Graphic, Ghanaian Times, Today Newspaper, Spectator,* and *Finder*), and general news outlets (*Ghana News Agency, Ghanaweb, Modern Ghana, News Ghana,* and *Ghana Today*). These media outlets were selected for the present study because they have strong online presence in Ghana. We complemented the search of these selected local media outlets by searching Google News™ to identify additional relevant media reports from Ghana.

English is the formal language of Ghana and the language for all written media contents in the country. Potentially eligible news reports on CPSA available between January 2000 and March 2019 were retrieved from the online portal of each selected media outlet by entering keywords and combinations of search terms and phrases (e.g. “rape”, “sexual abuse”, “man of God sexually assaults church member”).

### Criteria for Inclusion and Exclusion

**Fig. 1:**
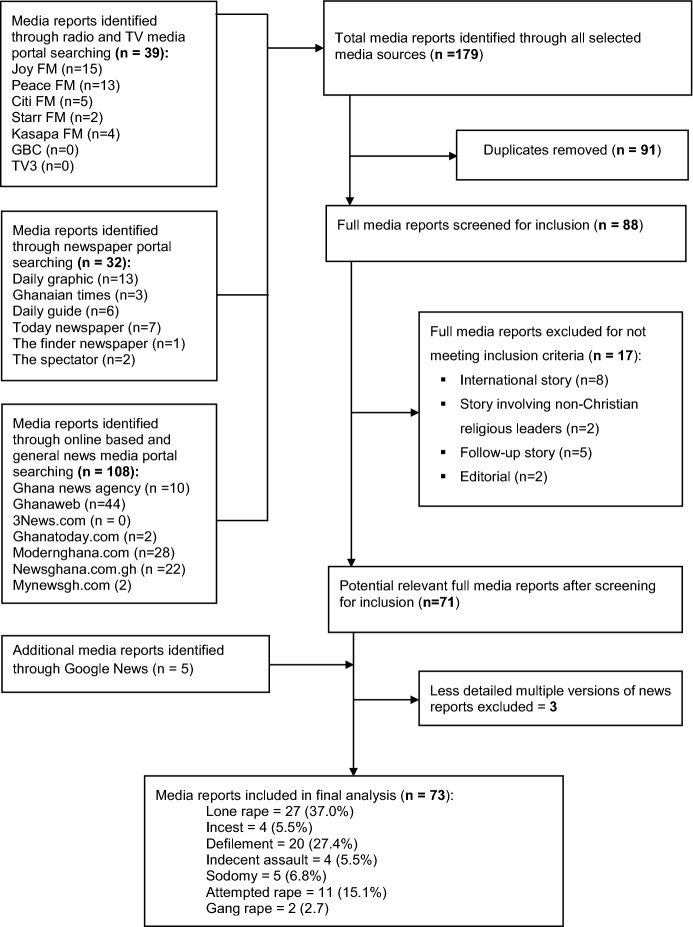
Flow chart of media portal search and news report extraction process

Local media reports on CPSA, and CPSA cases involving offenders whose leadership positions/roles have been explicitly indicated were included. However, news reports on CPSA that occurred outside Ghana, and CPSA reports involving non-Christian clergy were excluded from the final set of eligible media reports analysed for this study. Also, potentially eligible reports that represented a follow-up to an earlier news, and editorial pieces were excluded. Figure. 1 shows the search and extraction procedure followed to access eligible media reports for this study.

### Data Extraction and Analysis

We applied the summative content analysis technique (Hsieh & Shannon, 2005) to the data. We, iteratively, independently, and collectively, read each of the final set of eligible media reports on CPSA retrieved for the study. Guided by the aim of the study, we designed a data extraction form to obtain relevant information from each included news report (e.g. age and gender of survivor and perpetrator, denomination and leadership role of perpetrator, and relationship between survivor and perpetrator). Considering the exploratory and atheoretical nature of this study, the analysis was driven by the data. We assigned numerical codes to the extracted relevant manifest quantitative characteristics regarding the survivors, perpetrators, and the offence. We entered the numerically coded data into the Statistical Package for Social Sciences (SPSS version 26.0 for Windows), where the computation and tabulation of proportions and frequencies were performed. Furthermore, we extracted and presented textual excerpts of the news reports to explore the latent contents of the data.

Ethical approval from an Institutional Review Board was not sought for this study, as the recruitment and involvement of human participants were not part of this study. However, in writing this paper, identifying information (e.g. names, specific names of churches, addresses) of survivors and perpetrators of CPSA has been anonymised to protect the ethical position of the study.

## Findings

The search for media reports on CPSA from January 2000 through March 2019 yielded 179 hits. Of these, 73 met the inclusion criteria for this study (see Fig. 1). The 73 media reports analysed involved 105 survivors and 77 perpetrators (see Table 1).

**Table 1:** Cross-tabulation of victim size and perpetrator size across sexual offences

	Size	Lone rape (*n* = 27)	Incest (*n* = 4)	Defilement (*n* = 20)	Indecent assault (*n* = 4)	Sodomy (*n* = 5)	Attempted rape (*n* = 11)	Gang rape (*n* = 2)
		*n* (%)	*n* (%)	*n* (%)	*n* (%)	*n* (%)	*n* (%)	*n* (%)
Survivor size (*n* = 105)	1	24 (88.9)	4 (100)	17 (85.0)	2 (50.0)	3 (60.0)	9 (81.8)	2 (100)
	2	3 (11.1)	–	1 (5.0)	1 (25.0)	–	1 (9.1)	–
	3	–	–	–	–	1 (20.0)	–	–
	4	–	–	–	–	1 (20.0)	–	–
	5	–	–	1 (5.0)	–	–	1 (9.1)	–
	7	–	–	–	1 (25.0)	–	–	–
	8	–	–	1 (5.0)	–	–	–	–
Perpetrator size (*n* = 77)	1	27 (100)	4 (100)	20 (100)	4 (100)	5 (100)	11 (100)	–
	2	–	–	–	–	–	–	1 (50.0)
	4	–	–	–	–	–	–	1 (50.0)

### Survivor Characteristics

As shown in Table 1, there were 105 survivors: 99 females (94.3%) and six males (5.7%). It is worth pointing out that although there were 105 survivors involved in the cases analysed, the media reports provided information (on the key characteristics described in this paper) for only 73 of the survivors (67 females [91.8%] and 6 males [8.2%]). Thus, the analysis provided in this paper is limited to the 73 cases for which information was available about the key characteristics of the survivors, perpetrators, and the sexual offence. Three (4.1%) of the survivors were identified as (female) patients with mental health problems living with their families. Overall, the survivors were aged between 3 and 50 years (mean = 17.7; SD = 7.6), with the majority (54.8%) within the age bracket of 10–19 years. Between 1.4 and 11% of the survivors were identified as students, whereas 11% were reported as employed. Twenty-three (31.5%) were identified as single, eight (11%) as married, but the marital status of many (56.2%) was not reported. Lastly, 56.2% (*n* = 41) of the survivors lived with their family as at the time of the abuse, whereas 8.2% (*n* = 6) lived alone. Table 2 shows the results of the cross-tabulations of the key characteristics of the survivors, perpetrators, and the offence of sexual abuse across the study period.

**Table 2:** Characteristics of the victims, perpetrators, and the offence

	Category	Overall	Lone rape	Incest	Defilement	Indecent assault	Sodomy	Attempted rape	Gang rape
		73 (100%)	27 (37.0%)	4 (5.5%)	20 (27.4%)	4 (5.5%)	5 (6.8%)	11 (15.1%)	2 (2.7)
		*n* (%)	*n* (%)	*n* (%)	*n* (%)	*n* (%)	*n* (%)	*n* (%)	*n* (%)
*Survivor characteristics*									
Gender	Female	67 (91.8)	27 (40.3)	4 (6.0)	20 (29.9)	3 (4.5)	0	11 (16.4)	2 (3.0)
	Male	6 (8.2)	0	0	0	1 (16.7)	5 (83.3)	0	0
Age (in years)	< 10	5 (6.8)	2 (40.0)	0	2 (40.0)	1 (20.0)	0	0	0
	10–19	40 (54.8)	11 (27.5)	4 (10.0)	18 (45.0)	1 (2.5)	5 (12.5)	0	1 (2.5)
	20–39	19 (26.0)	10 (52.6)	0	0	2 (10.5)	0	7 (36.8)	0
	40–59	1 (1.4)	1 (100)	0	0	0	0	0	0
	NR	8 (11.0)	–	–	–	–	–	–	–
Educational level	No formal education	1 (1.4)	1 (100)	0	0	0	0	0	0
	Basic school	8 (11.0)	3 (37.5)	1 (12.5)	3 (37.5)	0	1 (12.5)	0	0
	Second cycle school	6 (8.2)	4 (66.7)	0	1 (16.7)	1 (16.7)	0	0	0
	Tertiary	3 (4.1)	1	0	1	0	0	1	0
	NR	55 (75.3)	–	–	–	–	–	–	–
Employment status	Unemployed	18 (24.7)	4 (22.2)	3 (16.7)	9 (50.0)	1 (5.6)	0	0	1 (5.6)
	Employed	8 (11.0)	2 (25.0)	0	1 (12.5)	0	0	5 (62.5)	0
	NR	47 (64.4)	–	–	–	–	–	–	–
Marital status	Single	23 (31.5)	8 (34.8)	3 (13.0)	10 (43.5)	1 (4.3)	0	0	1 (4.3)
	Married	8 (11.0)	3 (37.5)	0	0	0	0	5 (62.5)	0
	Widowed	1 (1.4)	0	0	0	1(100)	0	0	0
	NR	41 (56.2)	–	–	–	–	–	–	–
Living arrangement	Live alone	6 (8.2)	2 (33.3)	0	0	1 (16.7)	0	2 (33.3)	1 (16.7)
	Live with family	41 (56.2)	14 (34.1)	4 (9.8)	14 (34.1)	2 (4.9)	3 (7.3)	3 (7.3)	1 (2.4)
	Other	2 (2.7)	1 (50.0)	0	1 (50.0)	0	0	0	0
	NR	24 (32.9)	–	–	–	–	–	–	–
*Perpetrator characteristics*									
Age (in years)	20–39	33 (45.2)	15 (45.5)	1 (3.0)	11 (33.3)	1 (3.0)	2 (6.1)	2 (6.1)	1 (3.0)
	40–59	8 (11.0)	2 (25)	0	1 (12.5)	0	1 (12.5)	4 (50.0)	0
	≥ 60	1 (1.4)	1 (100)	0	0	0	0	0	0
	NR	31 (42.5)	–	–	–	–	–	–	–
Marital status	Married	8 (11.0)	2 (25.0)	3 (37.5)	2 (25.0)	0	0	1 (12.5)	0
	NR	65 (89.0)	–	–	–	–	–	–	–
Denomination	Catholic	2 (2.7)	1 (50.0)	0	0	0	0	1 (50.0)	0
	Protestant	5 (6.8)	3 (60.0)	0	0	1 (20.0)	0	1 (20.0)	0
	Pentecostal-Charismatic	23 (31.5)	8 (34.8)	0	5 (21.7)	2 (8.7)	2 (8.7)	4 (17.4)	2 (8.7)
	Neo-prophetic	34 (46.6)	14 (41.2)	3 (8.8)	10 (29.4)	1 (2.9)	2 (5.9)	4 (11.8)	0
	Unknown denomination	9 (12.3)	1 (11.1)	1 (11.1)	5 (55.6)	0	1 (11.1)	1 (11.1)	0
Role	Head/senior pastor	56 (76.7)	22 (39.3)	3 (5.4)	14 (25.0)	3 (5.4)	4 (7.1)	8 (14.3)	2 (3.6)
	Associate/junior/branch head	8 (11.0)	2 (25.0)	0	2 (25.0)	1 (12.5)	1 (12.5)	2 (25.0)	0
	Self-styled pastor	9 (12.3)	3 (33.3)	1 (11.1)	4 (44.4)	0	0	1 (11.1)	0
*Offence characteristics*									
Victim-perpetrator relationship	Church member	25 (34.2)	11 (44.0)	0	8 (32)	2 (8.0)	2 (8.0)	2 (8.0)	0
	Non-church member	21 (28.8)	8 (38.1)	0	4 (19.0)	0	1 (4.8)	7 (33.3)	1 (4.8)
	Neighbour	9 (12.3)	1 (11.1)	0	5 (55.6)	1 (11.1)	1 (11.1)	0	1 (11.1)
	Family member	10 (13.7)	3 (30.0)	4 (40.0)	2 (20.0)	0	0	1 (10.0)	0
	NR	8 (11.0)	–	–	–	–	–	–	–
Survivor’s problem	Infertility	1 (1.4)	1 (100)	0	0	0	0	0	0
	Marital problems	2 (2.7)	1 (50.0)	0	0	0	0	1 (50.0)	0
	Work problems	2 (2.7)	0	0	0	0	0	2 (100)	0
	Ill health	14 (19.2)	3 (21.4)	0	3 (21.4)	2 (14.3)	1 (7.1)	5 (35.7)	0
	Curses/diabolical problems	17 (23.3)	11 (64.7)	1 (5.9)	2 (11.8)	0	1 (5.9)	1 (5.9)	1 (5.9)
	NR	37 (50.7)	–	–	–	–	–	–	–
Compliance technique	Oracle-assigned-intercessor approach	34 (46.6)	18 (52.9)	0	8 (23.5)	1 (3.0)	1 (3.0)	6 (17.6)	0
	Drugging	6 (8.2)	3 (50)	0	2 (33.3)	0	0	0	1 (16.7)
	Blitz approach	20 (27.4)	3 (15.0)	3 (15.0)	8 (40.0)	1 (5.0)	1 (5.0)	3 (15.0)	1 (5.0)
	Multiple techniques	4 (5.5)	2 (50.0)	0	1 (25.0)	1 (25.0)	0	0	0
	NR	11 (15.1)	–	–	–	–	–	–	–
Location of the offence	Victim’s home	8 (11.0)	5 (62.5)	0	1 (12.5)	0	0	2 (25.0)	0
	Perpetrator’s home	45 (61.6)	15 (33.3)	3 (6.7)	15 (33.3)	3 (6.7)	3 (6.7)	5 (11.1)	1 (2.2)
	Isolated location	3 (4.1)	0	0	2 (66.7)	0	1 (33.3)	0	0
	Hotel/guest house	5 (6.8)	2 (40.0)	0	0	0	0	3 (60.0)	0
	Perpetrator’s office	2 (2.7)	1 (50.0)	0	0	1 (50.0)	0	0	0
	NR	10 (13.7)	–	–	–	–	–	–	–
Time of the offence	Morning	8 (11.0)	5 (62.5)	1 (12.5)	0	0	0	2 (25.0)	0
	Afternoon	5 (6.8)	2 (40.0)	0	2 (40.0)	0	0	1 (20.0)	0
	Night	22 (30.1)	7 (31.8)	1 (4.5)	8 (36.4)	1 (4.5)	1 (4.5)	4 (18.2)	0
	NR	38 (52.1)	–	–	–	–	–	–	–
Informant of police	Victim	16 (21.9)	7 (43.8)	0	0	0	0	8 (50.0)	1 (6.3)
	Victim’s family member	38 (52.1)	14 (36.8)	4 (10.5)	14 (36.8)	2 (5.3)	3 (7.9)	0	1 (2.6)
	Victim’s neighbour	1 (1.4)	1 (100)	0	0	0	0	0	0
	Another person	2 (2.7)	1 (50.0)	0	0	0	1 (50.0)	0	0
	NR	16 (21.9)	–	–	–	–	–	–	–
Time lag to disclosure	Same day	13 (17.8)	4 (30.8)	0	1 (7.7)	1 (7.7)	1 (7.7)	5 (38.5)	1 (7.7)
	Within one week	13 (17.8)	8 (61.5)	0	2 (15.4)	0	0	2 (15.4)	1 (7.7)
	After one month	14 (19.2)	4 (28.6)	2 (14.3)	7 (50.0)	0	1 (7.1)	0	0
	After one year	1 (1.4)	0	0	1 (100)	0	0	0	0
	NR	32 (43.8)	–	–	–	–	–	–	–
Legal outcome for perpetrator	In police custody/being investigated	28 (38.4)	4 (14.3)	1 (3.6)	9 (32.1)	3 (10.7)	2 (7.1)	8 (28.6)	1 (3.6)
	Being prosecuted	20 (27.4)	10 (50.0)	2 (10.0)	4 (20.0)	0	2 (10.0)	2 (10.0)	0
	Jailed	16 (21.9)	4 (25.0)	1 (6.3)	7 (43.8)	1 (6.3)	1 (6.3)	1 (6.3)	1 (6.3)
	At large/wanted by police	7 (9.6)	7 (100)	0	0	0	0	0	0
	NR	2 (2.7)	–	–	–	–	–	–	–
Jail term	2–9 years	3 (4.1)	0	0	1 (33.3)	1 (33.3)	0	1 (33.3)	0
	10–20 years	12 (16.4)	4 (33.3)	1 (8.3)	6 (50.0)	0	0	0	1 (8.3)
	25 years	1 (1.4)	0	0	0	0	1 (100)	0	0
Mental health & medical outcome for survivor	Emotional pain and trauma	8 (11.0)	5 (62.5)	1 (12.5)	2 (25.0)	0	0	0	0
	Gynaecological injuries and pain	12 (16.4)	4 (33.3)	1 (8.3)	5 (41.7)	0	1 (8.3)	1 (8.3)	0
	Pregnancy	5 (6.8)	1 (20.0)	0	4 (80.0)	0	0	0	0
	Infections	3 (4.1)	1 (33.3)	0	0	0	2 (66.7)	0	0
	NR	45 (61.0)	–	–	–	–	–	–	–

*NR* Not reported

### Perpetrator Characteristics

As shown in Table 1, although there were 77 all-male perpetrators involved in the 73 cases analysed, the included media reports provided information about the key characteristics of 73 perpetrators. Again, of the 77 perpetrators, two groups—made up of two and four members respectively—were found guilty of gang rape (see Table 1). As shown in Table 2, most of the perpetrators were aged between 20 and 39 years, were affiliated with neo-prophetic denominations (46.6%; *n* = 34), and were reported as head/senior pastors (76.7%; *n* = 56). Only 11% (*n* = 8) were reported as married, whereas the marital status of the majority (89%; *n* = 65) was not reported.

### Offence Characteristics

As indicated in Table 2, the 73 media reports analysed involved seven categories of sexual offences: lone rape (37.0%; *n* = 27), incest (5.5%; *n* = 4), defilement (27.4%; *n* = 20), indecent assault (5.5%; *n* = 4), sodomy (6.8%; *n* = 5), attempted rape (15.1%; *n* = 11), and gang rape (2.7%; *n* = 2). It is noteworthy that the news reports included in this study stated explicitly that the perpetrators were charged with or found guilty of these offences. Law enforcement agencies in Ghana make their determination of the offence based on the criminal code of Ghana^1^ (Act 29, 1960). Furthermore, in terms of survivors, these seven sexual offences were distributed across gender as follows: lone rape (*n* = 27; female = 100%), incest (*n* = 4; female = 100%), defilement (*n* = 20; female = 100%), indecent assault (*n* = 4; female = 75%; male = 24%); sodomy^2^ (*n* = 5; male = 100%), attempted rape (*n* = 11; female = 100%), and gang rape^3^ (*n* = 2; female = 100%).

### Survivor–Perpetrator Relationship and Survivor’s Problem

As shown in Table 2, some of the survivors (34.2%; *n* = 25) were identified as church members of the perpetrators; many of the survivors who were congregants of the perpetrators reported lone rape (44%; *n* = 11). Also, most of the survivors were reported to have first approached the perpetrators with needs (for help) related to curses/diabolical problems (23.3%; *n* = 17), and ill health (19.2%; *n* = 14). For instance, in one news report involving a female survivor aged 22 years, it was mentioned that:

Assistant Commissioner of Police, [*name anonymised*] … made it known to the press [at *name of place anonymised*] that… Pastor [*name anonymised*] admitted having sexual intercourse with the girl without her consent, during police interrogations. […] The victim, a member of the church, who was sick went to the pastor (in his house) for healing prayers. In the course of the prayers the pastor touched the mouth of the girl and all of a sudden, she fell floppy […] The victim woke up after about 15 minutes and saw that her pant was loose, and her private parts soaked with semen, an indication that the pastor had had sexual intercourse with her (Graphic Online, 2013).

In another news report:

The victim (female, 20 years old) […] approached [*the perpetrator, aged 39 years*], [*name of perpetrator anonymised*], to help her break a blood covenant^4^ she had with her boyfriend. [*the perpetrator*] agreed to help her and asked [*name of victim anonymised*] to meet him […] in a hotel […]. She complied and met [*the perpetrator*] in one of the rooms where he forcibly had sex with her (Myjoyonline, 2013).

In another case involving a 15-year-old mental health female patient:

A 25-year old Pastor [*who pleaded guilty to defilement*] … impregnated a school drop-out, [*who was also*] a mentally ill patient … The police inspector said, the pastor who was praying for the 15-year old victim, told the girl one night that he had a vision to have sexual intercourse with her in order [*for her*] to be healed. The police officer said since then the "Man of God" had frequent sex with the girl without opposition until she became pregnant (Ghana News Agency, 2009).

### Compliance Technique

The perpetrators used three main singular approaches to get their victims to comply: *oracle-assigned-intercessor* approach, drugging, and use of a blitz approach.^5^ Others used multiple techniques, where two or all the three singular approaches were combined to obtain compliance from the victim. However, predominantly, the perpetrators employed the *oracle-assigned-intercessor* approach (46.6%; *n* = 34) to elicit compliance from their victims. In this study, we used *oracle-assigned-intercessor* approach to denote the situation where the perpetrator directly approaches their potential victim (or indirectly through their relative) with, usually, an unpleasant “divine revelation or prophesy” concerning the life or future of the potential victim, but indicates (directly or indirectly) that he—the perpetrator—is the only one “assigned or designated” by divine authority to intercede to avert the unpleasant oracle, but in the process sexually abuses the unsuspecting congregant or client. For example, in a news report involving an 18-year-old female survivor:

The pastor confided in the victim’s mother after church service on Sunday […] that God had revealed to him through a vision that her daughter was possessed with an evil spirit. He requested the woman to allow the girl [to] come for deliverance service on Tuesday. On that Tuesday, the mother of the victim asked her daughter to go for [the] service, but when the victim got to the church, the pastor requested that they go to his house for the said deliverance to be carried out and the victim complied. […]. The pastor then ordered the victim to lie down and remove her dress, while he rubbed oil on her navel and parts of her abdomen, […] at that point he grabbed the victim and forcefully kissed her (Dailyguide Africa, 2016).

In another sombre media report, there were four boys (two 14-year-olds, and two 16-year-olds) who were reported to have been raped by a pastor as follows:

Presenting the facts of the case in court, Assistant Superintendent of police [*name anonymized*] disclosed that […] the pastor told the boy that he would be impotent in the future, and therefore, invited him to his house for prayers, and when the boy honoured the invitation, he had anal sex with him. […] In the same month, another boy fell ill and visited the prayer camp, and the accused, after praying for and anointing him with oil, […] took him [*the boy*] to his room and sodomised him. The prosecution said [*that*], in the following two months, a third boy visited the prayer camp and was told by the accused that he was possessed by demonic spirits which would make it difficult for him to have children in the future. […] The boy was then told by the accused that the only way to cast out the demons was for him to have anal sex with him. He convinced the boy and had it [sex] with him. The accused invited another boy who was passing by the prayer camp and told him that he was being spiritually chased by girls and that would affect his studies. He asked the boy to see him later in the evening for prayers. When the boy turned up at the prayer camp, the accused, after praying for him, […] ordered the boy to strip naked and told him that he needed to have anal sex with him, to exorcise the evil spirits. The accused, then abused the boy sexually (Peacefmonline, 2015).

### Temporal Characteristics

Most of the CPSA cases took place in the homes of the perpetrators (61.6%; *n* = 45), and at night (30.1%; *n* = 22). Also, as shown in Table 3, many of the cases (35.6%; *n* = 26) were reported in the Greater Accra region, while 4.1% (*n* = 3) were reported each from the Volta region and the Western region (see Table 3).

**Table 3:** Regional distribution of media reported clergy-perpetrated sexual abuse cases

Region	Frequency	Per cent
Greater Accra Region	26	35.6
Ashanti Region	16	21.9
Central Region	15	20.5
Brong-Ahafo Region	5	6.8
Eastern Region	5	6.8
Volta Region	3	4.1
Western Region	3	4.1
Total	73	100

### Legal and Medical Implications of Abuse

Less than one-fourth of the 73 CPSA cases analysed were reported to the police on the same day the offence took place (17.8%; *n* = 13). More than half of the cases (52.1%; *n* = 38) were reported to the police by a family member of the survivor (e.g. mother, father, husband, etc.), while 21.9% (*n* = 16) of the cases were reported directly to the police by the survivors themselves (see Table 2). More pointedly, as shown in Table 2, during the period when the media reports were published, many of the perpetrators (38.4%; *n* = 28) were in police custody/being investigated, whereas 27.4% (*n* = 20) were being prosecuted at the law court. Also, 21.9% (*n* = 16) of the perpetrators who were found guilty by the law court were jailed between two and 25 years (in most cases, with hard labour). The majority (75%; *n* = 12) of these 16 perpetrators were jailed between 10 and 20 years, with 50% (*n* = 6) being guilty of defilement.

Even though the causal attributions by the perpetrators were not reported, in one of the cases where the perpetrator was found guilty and jailed by a court of competent jurisdiction, the court described the conduct of the perpetrator as:

A disgrace to all men of God and it is a betrayal of Christian principles. The accused is being convicted and sentenced to serve as a deterrent to all so-called prophets who sexually assault innocent [girls and] women and people's wives (Ghanaweb, 2007).

One potential interpretation of this comment could be that, although CPSA is often an individual, private act, it is criminal and invariably damages the collective image and nobility of the Christian pastoral community and clergy.

Regarding the mental health and medical outcome for the survivors (as shown in Table 2), some reported emotional pain and trauma (11%; *n* = 8); others reported gynaecological injuries and pain (16.4%; *n* = 12), particularly, the survivors of defilement (41.7%; *n* = 7), while 4.1% (*n* = 3) got infected with a sexually transmitted disease as a result of the offence (e.g. HIV/AIDS).

## Discussion

This study represents the first and pioneering attempt at providing a systematic evidence on the reality of CPSA in Ghana. Some of the key findings warrant discussion, as they have implications for practice and future research regarding the phenomenon.

### Survivors and Perpetrators of CPSA

This study shows that most (91.8%) of the survivors of CPSA were females, of which more than half (54.8%) were adolescent girls aged between 10 and 19 years. In contrast, the perpetrators were all males, mainly aged between 20 and 39 years. These findings are not surprising, as they support evidence from Ghana (Boakye, 2009; Boateng & Lee, 2014; Quarshie et al., 2017, 2018) and are consistent with the global literature (UNICEF, 2014; WHO, 2002) that, mainly, young males carry out various forms of sexual violence and abuse against women and girls, regardless of the social, organisational, or institutional context, although young boys are not entirely exempted from sexual exploitation victimisation (Adjei & Saewyc, 2017; Quarshie, 2021; UNICEF, 2020).

### Offence Characteristics

An interesting finding of this study is that most of the survivors presented with problems related to ill health and curses/diabolical problems. Consistent with the African cosmological ideas of causality and religious interventionism, the Ghanaian worldview holds that the aetiology of ill health—including mental disorders—is rooted profoundly within the cultural beliefs related to cosmic forces and evil machinations. It is believed that evil forces partake in the affairs of humans, creating discomfort and illness, and manipulating people to engage in wrong deeds (Assimeng, 2007; Danquah, 1982; Salifu Yendork et al., 2019). Prayers and other religious rituals including divination practices are believed to be the means by which the activities of cosmic forces and evil machinations can be prevented and removed from the affairs of a people (Assimeng, 2007). In this vein, religious leaders in Ghana tend to represent frontline care workers called on by both congregants and non-congregants for help to ensure physical, mental, and spiritual well-being and safety (Arias et al., 2016; Nukunya, 2016; Osafo, 2016). However, there are reports—including the evidence in this study—of religious zealotry of some religious leaders and groups, which leads to various forms of abuse of congregants and supplicants in Ghana (Badu et al., 2019; Osafo, 2016; Ssengooba et al., 2012).

Another interesting finding of our study is that, predominantly, the perpetrators occupied head/senior pastor positions, were mainly in Neo-prophetic denominations, and made use of the oracle-assigned-intercessor approach to elicit compliance from the survivors. The possible linkage of these three elements is not entirely surprising—it could be reflecting the possibility that the perpetrators of CPSA capitalise on three key factors. The first is that, generally, Ghanaians hold absolute trust in religious leaders (Assimeng, 2010). Typically, regardless of religious affiliation, the Ghanaian has “unquestioning acquiescence to the dictates of men of influence, and rules and regulations … [and] fetish worship of authority and charismatic leaders, and of doing things coming from above” (Assimeng, 2007, pp. 114–115). Thus, it appears that a potential perpetrator of CPSA who is charismatic, with a Neo-prophetic orientation (prophetism), and who “gets assigned by the oracle” to intervene on behalf of a supplicant is likely to get at their victims.

Generally, Neo-prophetic churches are gaining popularity and flourishing within sub-Saharan Africa mainly due to their emphasis on healing and exorcism in their teachings; they represent a new way of prophetism, in keeping with their Pentecostal theological orientation (Omenyo, 2011; Osafo et al., 2015). As observed by Omenyo and Arthur (2013, p. 51) “their predominant feature is the prophetic ministry, a feature that attracts a large clientele to them”. Neo-prophetic churches have been commonly described in Ghana as “one-man churches”, as they are often run and built around a single charismatic pastor, who is also usually the founder and head pastor (Roberts, 2009). Typically, Neo-prophetic churches have no affiliation with the GCBC, CCG, or GPCC, and many of them are not duly registered and incorporated as required by the laws of Ghana.

The second factor is that Christians are admonished by the Bible (2 Chronicles 20:20—New King James Version) to “Believe in the LORD your God, and you shall be established; believe His prophets, and you shall prosper”. The implication of this biblical recommendation is that a congregant who wants to prosper will heed the requests of their prophets, as the congregant considers the teachings of the Bible seriously (Agazue, 2016).

Thirdly, as a leader, a priest in the Christian congregation wields spiritual power (Ayodele, 2019; Doyle, 2009; Harper et al., 2020; Rashid & Barron, 2019). In other words, it is possible that a priest’s position as a spiritual leader, who is commonly perceived by the congregants as being fundamentally unique from a lay person and likened to Christ (Ayodele, 2019; Doyle, 2009; Harper et al., 2020; Rashid & Barron, 2019), might have influenced the compliance of the survivors in the cases of CPSA analysed in the current study.

Furthermore, the use of a blitz approach is not surprising, as most of the perpetrators were early adult males (aged between 20 and 39 years), with the survivors being children and adolescent girls aged 20 years and younger. Relatively, the perpetrators are likely to have higher physical strength to overpower their victims (Berk & Meyers, 2015). Considering that the perpetrators were relatively older than the survivors, the perpetrators were also likely to have wielded some social power over the survivors, as younger people in Ghana—and across Africa in general—are exhorted to obey adults and be submissive to them, in order to win the appreciation and goodwill of their adults (Gyekye, 2003; Nukunya, 2016).

Consistent with existing evidence (Agazue, 2016; Denney et al., 2018; Firestone et al., 2009; Terry et al., 2011), the present study has shown that the location of the offence was mostly the perpetrators’ homes. Given the humanitarian (and sometimes on-call) nature of their work, generally, religious leaders living in their own homes, manses, or mission houses in Ghana tend to operate an open-door policy, where both congregants and non-congregants can call on a religious leader for support. Others also come in to make donations to support church work. It, thus, appears that perpetrators of CPSA take advantage of this seeming friendly and non-threatening environment to access and abuse their unsuspecting victims.

Taken together, the reported cases of CPSA mostly occurred in the Greater Accra, Ashanti and Central regions of Ghana. Besides most people in these regions identifying as Christians (Ghana Statistical Service, 2013), many Charismatic-Pentecostal and Neo-prophetic churches (compared to historical mainline mission churches) are often established within the capital cities of these regions, where educated and upwardly mobile youth and the middle class who can support the church financially are often located (Benyah, 2018; Gifford, 1994; Roberts, 2009). Also, the strong presence of the media (i.e. television and radio) in these regions and cities is harnessed, particularly, by Neo-prophetic ministries to promote their activities and programmes (Benyah, 2018). Furthermore, rapid population growth and rapid urbanisation in these regions have placed high pressure on professional health care services; there is high (youth) unemployment, and generally, there is a breakdown of the traditional ways of marital conflict resolution in urban areas (Ghana Health Service, 2017; Nukunya, 2016). To help government address some of these problems, religious and faith-based groups and communities carry out complementary intervention and prevention programmes to support vulnerable people and resource-poor local communities (Osafo et al., 2019; Taylor et al., 2000). However, it appears that perpetrators of CPSA take undue advantage of the vulnerability of persons presenting with the need for support against these health, social, and economic challenges by abusing them sexually.

An encouraging finding of this study though is that most of the CPSA cases (78.1%) were reported to the police and the time lag to disclosure was between the same day of the offence and a few weeks after the offence (54.8%). Additionally, most of the survivors (56%) in this study were reported as living with their families and the majority of the CPSA cases (52.1%) were reported to the police by a member of the survivor’s family. Put together, these findings could be in support of recent evidence from Ghana that survivors of sexual abuse and significant others around them are not only becoming aware of the importance of reporting sexual offences to the police but are also heeding the advice of seeking help from formal support institutions—including the police—particularly, for survivors of sexual abuse (Osam, 2004; Quarshie et al., 2017, 2018).

### Implications of Study

The worrying nature of the evidence presented in this study underscores the need for further studies on CPSA in Ghana. Future studies could explore the motivations of convicted offenders and the experiences of survivors, to obtain evidence that could potentially inform intervention and prevention efforts and programmes. Churches could formulate and enforce policies regulating (or possibly prohibiting) leaders from offering one-to-one pastoral support to (young) supplicants in private spaces. The mass media and the various associations of Christian denominations and ecumenical bodies could collaborate to provide public education on the need (for both congregants and non-congregants) to be cautious of receiving one-to-one pastoral support off-site and on-site under compromising circumstances. Relatedly, parents and families must avoid leaving their minors alone with their pastors while receiving spiritual support.

The current study has reported sexual abuse perpetrated by only Christian clergymen. However, to obtain a more comprehensive understanding and a clearer sense of the extent of sexual abuse perpetration by religious leaders in Ghana, future studies could focus on examining the phenomenon among non-Christian religious groupings in the country—for example, Islam, African Traditionalists, and other indigenous religious groups. A recent study from the UK has found that some Muslim clerics also perpetrate sexual abuses against congregants (Chowdhury et al., 2021).

This study further highlights the need for government to collaborate with the associations of churches and ecumenical bodies in Ghana (i.e. GCBC, CCG and GPCC) to strengthen and streamline the recruitment, training, and leadership structures of Christian and other religious denominations in the country. The evidence of this study shows that most of the problems presented by the survivors of CPSA were needs requiring broader government social interventions (e.g. unemployment, healthcare). Thus, an overarching implication of this study is the need for government to expand social intervention projects and programmes related to the provision of accessible and affordable—mental—health care (e.g. by broadening the coverage of the National Health Insurance Scheme), tackling youth unemployment, and teaching interpersonal and marital conflict resolution skills and family values to married and potential couples.

### Study Limitations

The findings and conclusions drawn by this study must be considered cautiously in the light of the limitations associated generally with the approach and data sources used. Recent evidence suggests that besides predatory clergymen using their spiritual authority and organised institutional power to intimidate and prevent their victims from reporting the abuse, the organisational and management structures and the code of congregant conduct of many historical mainline mission churches, particularly, the Roman Catholic Church, prevent survivors of CPSA from reporting the offence (Ayodele, 2019; Harper et al., 2020; Rashid & Barron, 2019). Thus, on the one hand, it is possible that relative to Pentecostal, Charismatic, and Neo-prophetic churches, many cases of CPSA go unreported in most historical mainline mission churches in Ghana (e.g. Catholic, Methodist, and Presbyterian churches) and may not attract media attention. On the other hand, it is also critical to indicate that one needs to be cautious when attempting to generalise our findings to all Christian denominations and to all clergy—particularly, Neo-prophetic churches and their pastors. There are still non-abusive clergy and Christian denominations that abhor and courageously deal with sexual abuse perpetration by clergy as a criminal issue.

Like all sexual offences, not all cases of CPSA are reported in the media, and we did not search the online portals of all local media outlets in Ghana for this study. As observed by previous studies on media contents of sexual abuse, often, the sensational and extreme cases of sexual offences are reported, with many important information missing—as shown by the many zeros in the Tables presented in this paper (e.g. Quarshie et al., 2017, 2018). This makes generalisation of the findings difficult. Notably also, media reports about CPSA can be subject to selection biases of the media outlet or journalist who reports the stories. While the decision to report CPSA-related news stories is determined by the interests of the media outlet, news about CPSA are likely to contain untruths, which may not reflect the full facts. Future studies using media content analysis approach could consider following up the perpetrators, survivors, and organisations that work directly with cases of CPSA, in order to supplement the data drawn from media contents.

Relatedly, the reporting style of the Ghanaian media can be problematic in terms of CPSA. The explicit reporting of the identity information (e.g. names) of survivors and perpetrators can (inadvertently) induce stigma, shame, and other psychological distress, which can militate against recovery, particularly, among survivors and their families (Quarshie et al., 2018). The detailed description of related situational features of the offence (e.g. graphic depiction of the methods used for the act) in the media could upset readers but also induce modelling in potential perpetrators. Therefore, media outlets in Ghana and key stakeholders should consider the development and adoption of sensitive, ethically sound, and culturally relevant guidelines for reporting CPSA (and other sexual offences) in the Ghanaian media.

Lastly, it is imperative to emphasise that this media content analysis did not draw attention to reports of the beneficial work that many religious churches/congregations are doing in Ghana, nor is it being asserted that all clergy are promoting irrationality and fear, for most clergy (in line with their theology and ecclesiastical doctrines) would, or at least should, advocate for positive and ethically responsible behaviour for both clergy and their congregational adherents.

## Conclusion

The evidence of the current study has shown that in Ghana, media reports indicate that CPSA predominantly occurs in Neo-prophetic denominations, often perpetrated by male pastors (aged between 29 and 39 years) occupying religious positions of trust, with adolescent girls aged 10–19 years as the predominant survivors. To elicit compliance, the perpetrators convinced the survivors that they (the perpetrators) were “designated by God” as the intercessors to help find solutions to the problems of the survivors. In other instances, the perpetrators used violence-based approaches to overpower the survivors. Whereas some of the perpetrators were reported to be in police custody, most of the survivors reported various negative health outcomes (e.g. trauma, gynaecological injuries, sexually transmitted infections) consequent upon the abuse. Although it might be difficult to convince congregants not to believe strange prophecies from their religious leaders, it might be helpful for congregants to reflect on and apply rationality, rather than emotions and fear, to prophetic claims given to them by their church leaders. Perhaps, the evidence of this study also alerts families and (young) female congregants to see as a red flag of CPSA when their religious leaders make incredible requests or suggest rituals/exorcisms involving nudity or sensual acts. Broadly, this study underscores the need for strengthening and streamlining the recruitment, training, and leadership structures of Christian (and non-Christian) denominations in Ghana.

## Footnotes

1 The Criminal Code of Ghana (Act 29, 1960) defines rape, incest, defilement, indecent assault as follows:

- Sect. 98: **Rape**—Is the carnal knowledge of a female of not less than sixteen years without her consent.
- Sect. 105 (1): **Incest**—A male of not less than sixteen years of age who has carnal knowledge of a female whom he knows to be his grand-daughter, daughter, sister, mother or grandmother commits a criminal offence and is liable on summary conviction to a term of imprisonment of not less than three years and not more than twenty-five years.
- Sect. 101 (1 & 2): **Defilement**—(1) Defilement is the natural or unnatural carnal knowledge of a child under sixteen years of age; (2) Whoever naturally or unnaturally carnally knows a child under sixteen years of age, whether with or without the consent of the child, commits a criminal offence and is liable on summary conviction to a term of imprisonment of not less than seven years and not more than twenty-five years.
- Sect. 103 (2): **Indecent assault** – A person commits the criminal offence of indecent assault if, without the consent of the other person that person.
- Forcibly makes a sexual bodily contact with the other person, or.
- Sexually violates the body of that other person, in a manner not amounting to carnal knowledge or unnatural carnal knowledge.

2 *Sodomy* The crime of defilement where the victim is a male (Ghana Police Service, 2015).

3 *Gang rape* “Rape of a person by two or more perpetrators” (Jewkes, Sen, & Garcia-Moreno, 2002, p. 149).

4 Blood covenant is a practice where lovers inflict cuts on a finger each and then bring the wounded fingers together for the blood from one partner to mix with the other partner's to signify a promise that they would never break their relationship till death. Those engaged in the practice believe that the one who would break the relationship would die prematurely. Religiously, this practice is believed to have dire spiritual implications, whereas medically, it has been found as one of the pathways to contracting HIV/AIDS, particularly among young people in Ghana (Ghana News Agency, 2003.). Accessed from: https://www.modernghana.com/news/36642/students-in-blood-covenant-despite-aids-prevalence.html#

5 In the blitz approach, the perpetrator applies sudden violence and overpowers the survivor (e.g., by gaging, hitting, dragging, etc.).

## Declarations

### Data availability

The datasets used and/or analysed during the current study are available from the corresponding author on reasonable request.
